# Automated detection and enumeration of marine wildlife using unmanned aircraft systems (UAS) and thermal imagery

**DOI:** 10.1038/srep45127

**Published:** 2017-03-24

**Authors:** A. C. Seymour, J. Dale, M. Hammill, P. N. Halpin, D. W. Johnston

**Affiliations:** 1 Division of Marine Science and Conservation, Nicholas School of the Environment, Duke University Marine Laboratory, 135 Duke Marine Lab Rd, Beaufort, NC 28516, USA; 2 Peches et Oceans Canada/Fisheries and Oceans Canada. Institut Maurice-Lamontagne/Maurice Lamontagne Institute, C.P. 1000/P.O. Box 1000 850 Route de la Mer Mont-Joli, QC, G5H 3Z4, Canada.

## Abstract

Estimating animal populations is critical for wildlife management. Aerial surveys are used for generating population estimates, but can be hampered by cost, logistical complexity, and human risk. Additionally, human counts of organisms in aerial imagery can be tedious and subjective. Automated approaches show promise, but can be constrained by long setup times and difficulty discriminating animals in aggregations. We combine unmanned aircraft systems (UAS), thermal imagery and computer vision to improve traditional wildlife survey methods. During spring 2015, we flew fixed-wing UAS equipped with thermal sensors, imaging two grey seal (*Halichoerus grypus*) breeding colonies in eastern Canada. Human analysts counted and classified individual seals in imagery manually. Concurrently, an automated classification and detection algorithm discriminated seals based upon temperature, size, and shape of thermal signatures. Automated counts were within 95–98% of human estimates; at Saddle Island, the model estimated 894 seals compared to analyst counts of 913, and at Hay Island estimated 2188 seals compared to analysts’ 2311. The algorithm improves upon shortcomings of computer vision by effectively recognizing seals in aggregations while keeping model setup time minimal. Our study illustrates how UAS, thermal imagery, and automated detection can be combined to efficiently collect population data critical to wildlife management.

Estimating the abundance of animal populations is essential for both fundamental and applied wildlife ecology^1^,^2^. A good understanding of the abundance or density of a species or population within an ecosystem is required to elucidate its ecological roles within a community^3^, and to determine how best to manage human interactions with animal populations^2^.

Generating abundance information about a species usually relies on human survey effort to either census a population, or estimate its numbers through a statistically robust sampling program. In many cases, these efforts employ real-time visual^4^ or post-processed remote sensing surveys^5^ that allow for direct or indirect enumeration of individual animals or their indicators, such as tracks, scat or sounds^2^.

Aerial surveys are particularly useful for species that make use of predictable habitats for resting, mating, breeding, feeding or other social activities. This is especially true for marine species such as seabirds and pinnipeds that spend significant time at sea where they are cryptic and largely unavailable for visual or remotely-sensed detection. For these species, ground-based or aerial surveys at terrestrial/on ice aggregation sites provide for efficient abundance assessments.

Traditional surveys of many animal colonies are conducted using human-occupied helicopters or fixed-wing aircraft, which is risky^6^, costly for small study areas and can disturb animals during image collection^7^. The increasing resolution of satellite imagery has provided new opportunities to assess wildlife populations from space^8^. However satellite-derived methods have difficulty resolving smaller animals^9^, and are hampered by atmospheric interference from clouds and humidity. The use of unmanned aircraft systems (UAS) for wildlife population assessments can often reduce costs and risk to humans while providing extremely high resolution imagery of small species, overcoming the constraints of satellites and occupied aircraft^10^.

The use of UAS in terrestrial research applications is now wide-spread and transforming spatial ecological science^11^. These devices are used regularly to reduce costs and increase knowledge of key agricultural parameters through a combination of visible and multispectral imagery^12^. Small UAS are used to assess progress and environmental compliance in mining operations and increasingly to study the terrestrial habitats and aggregations of wildlife^13^. The use of UAS in marine science is also on the rise, with applications focused on coastal ocean processes, habitats and species. For example, UAS have been used to assess ocean temperatures^14^, ocean productivity^15^, and coastal geomorphology^16^. They have also been used to assess seagrass beds^17^, for shoreline habitat mapping and coastal erosion studies^16^, and to assess the abundance and health of marine vertebrates^18^,^19^.

While UAS surveys collect detailed information rapidly, they do not overcome existing data analysis bottlenecks. Specifically, manual counting of animals in imagery is time consuming and inefficient^20^. However, recent advances in automated counting and computer vision/machine learning approaches can help overcome these inefficiencies^20^. For example, automated detection and counting of birds^21^ and marsupials and mammals^22^,^23^ can speed up population assessments and, depending on the sensors used, real-time applications are possible. Thermal sensors are particularly useful for mammalian wildlife, which tend to emit thermal energy at 9–14 μm wavelengths that is detectable by a range of commercially available sensors^24^. Recent studies have capitalized on steep thermal gradients between mammalian targets and their backgrounds to identify, localize and count them^25^.

Like many terrestrial carnivores, coastal predators in North America were severely depleted during the 19^th^ and 20^th^ centuries. Subsequent conservation actions have enabled the recovery of many populations^26^. In eastern North America, grey seal (*Halichoerus grypus*) populations were once depleted through bounties and hunts^27^,^28^, and have subsequently made remarkable recoveries across much of their range^29^. As this species recovers and reassumes its roles in coastal ecosystems, there is a need for cost efficient and accurate abundance estimates to inform management^30^.

This paper illustrates the combination of (1) efficient UAS surveys, (2) thermal imaging and (3) a simple to use GIS-based workflow, to enhance the detection and enumeration of a marine predator at predictable terrestrial breeding colonies. Specifically, the present study estimates the abundance of grey seals at two breeding colonies in eastern Canada through automated counting of thermal imagery, classifying each individual seal as either an adult (>1 year old) or pup/young of the year (hereafter YOY, <1 year old). These counts are then compared to traditional human-generated counts of seals in both thermal and RGB imagery.

## Methods

### Study Location

**Figure 1:**
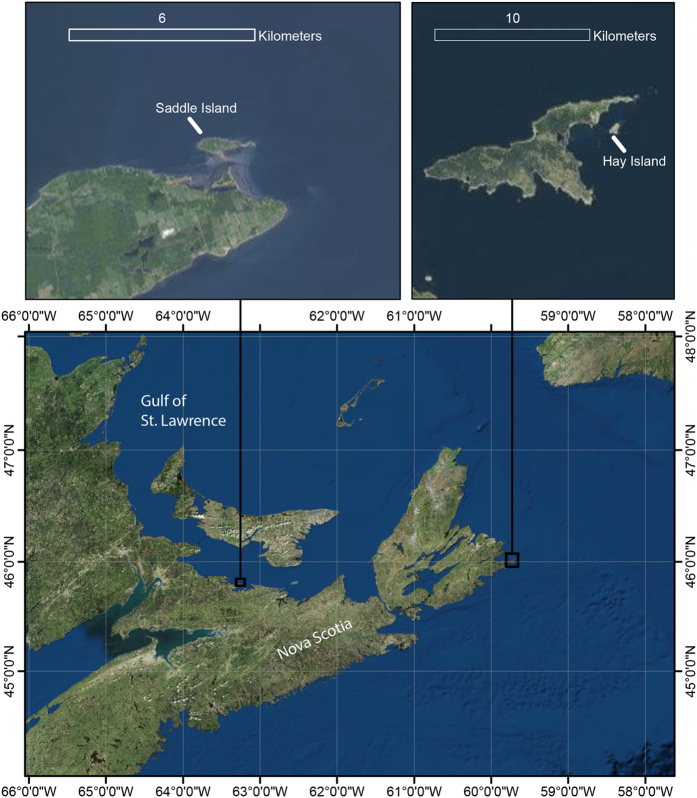
Locations of grey seal Halichoerus grypus breeding colonies on Saddle and Hay Island, Nova Scotia, Canada surveyed with unmanned aircraft systems (UAS) during January 29–February 2 2015. This map was created with ArcMap GIS software (version 10.4.1, Esri Inc.) using ArcMap’s World Imagery service layer. Service Layer Credits: Source: Esri, Digital Globe, GeoEye, Earthstar, Geographics, CNES/Airbus DS, USDA, USGS, AEX, Getmapping, Aerogrid, IGN, IGP, swisstopo and the GIS user community.

Surveys for adult and YOY grey seals were conducted during the breeding season (January 29 to February 2, 2015) at Hay Island (approximately 46.022593° N, −59.685304° W) and Saddle Island (approximately 45.814449° N, −63.251189° W), two breeding colonies in the Gulf of St. Lawrence, Nova Scotia, Canada (Fig. 1).

### Small Unmanned Aircraft System

All UAS Surveys were conducted with the senseFly eBee, a commercially available fixed wing UAS. These modular devices are light-weight foam airframes powered by a single brushless electric motor supplied by lithium polymer batteries. They have a wing-span of 96 cm, weigh 0.7 kg and are highly portable, fitting into a case capable of being carried as cabin baggage on airliners for transport.

The single engine and propeller are rear-mounted to ensure the safety of both the UAS and the people operating it. The aerodynamic profile allows the UAS to cruise at speeds of 36–57 km/h (10–16 m/s) and resist winds of up to 45 km/h (12 m/s). The UAS was pre-programmed in eMotion 2 software package (senseFly, Switzerland) to follow a 3-dimensional flight path guided by a precision GPS sensor, a high-resolution barometer, ground-sensing camera and wind-speed sensor. Failsafe logic within the autopilot was programmed to return the UAS to the landing zone if it experienced anomalies in sensor performance or extreme wind conditions. All flight data was telemetered to the operator over VHF frequencies in real-time. The instrument was launched by hand and recovered after either a linear approach/landing at a predetermined 5 m radius region.

### Sensors

Seal surveys were conducted at each colony using both a 12 megapixel RGB camera (Canon S110) and a 640 × 512-pixel thermal infrared camera (senseFly LLC, Thermomapper). This sensor is self-calibrating, with a marketed precision of 0.1 °C (senseFly LLC). Comparisons with ground-based temperature measurements indicate that it is accurate within 1 °C. RGB imagery was captured at a shutter speed of 1/2000^th^ of a second at 3 cm ground sampling resolution with a photo taken approximately every 3 seconds. Thermal imagery was captured at sub-second intervals and at 8 cm ground sampling resolution at each location.

### Seal Count Data

Images collected by the UAV were processed using Pix4d software to create RGB orthomosaics and thermal spatial indices (°C) of both colonies. All orthomosaics and thermal indices were corrected for any inconsistencies due to animal movement between adjacent images, exported as GeoTIFF files and then then imported into the iTag software package. Human analysts manually counted the number of seals and classified each as either an adult or a YOY. Temperature index GeoTIFFs were also imported into ArcMap GIS software (Version 10.4.1, ESRI Inc) for subsequent automated detection and counting of adult and YOY seals.

### Automated detection and Counting of seals

**Figure 2:**
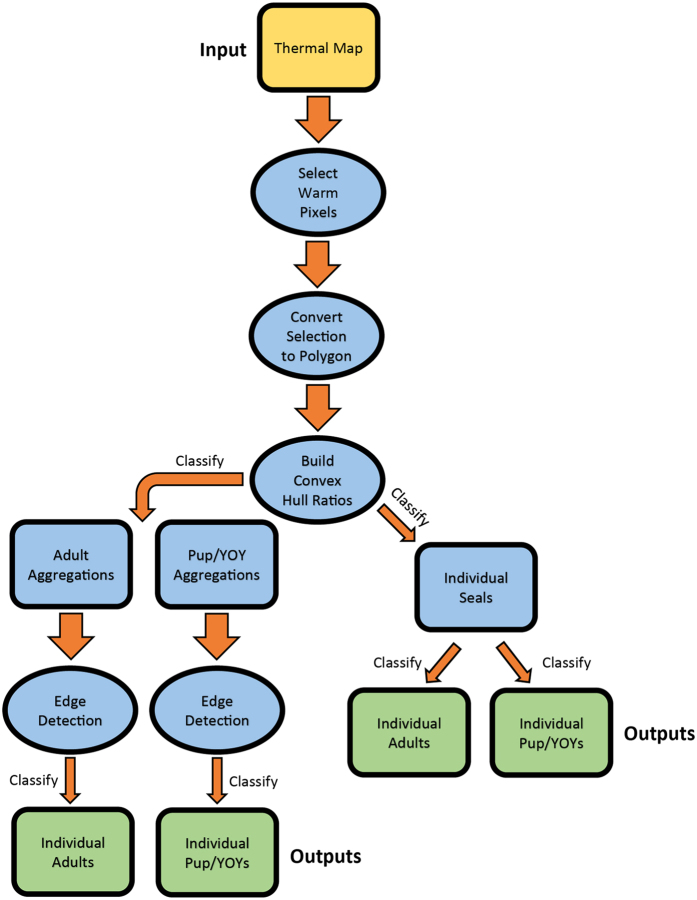
An overview of the seal detection model’s logical processes. Yellow icons are inputs, blue icons are intermediate processes and outputs, and green icons are final outputs.

The seal detection model was built in the ArcGIS model builder programming environment and was designed to scan thermal imagery, detect seals and count them. The tool used spectral thresholds and pixel cluster size sorting to detect grey seal adults and YOY, and integrated object recognition and high pass filtering (i.e. edge detection) to discriminate individuals within closely packed aggregations. The tool’s logical overview can be seen in Fig. 2, and the full tool script can be seen in appendix A of the Supplementary Materials.

### Methodological Details

Thermal and RGB mosaic spatial indices of Hay Island were first masked by excluding areas around the southern and eastern edge of the island where the ambient temperature of landscape features overlapped with the temperature of seals.

**Figure 3:**
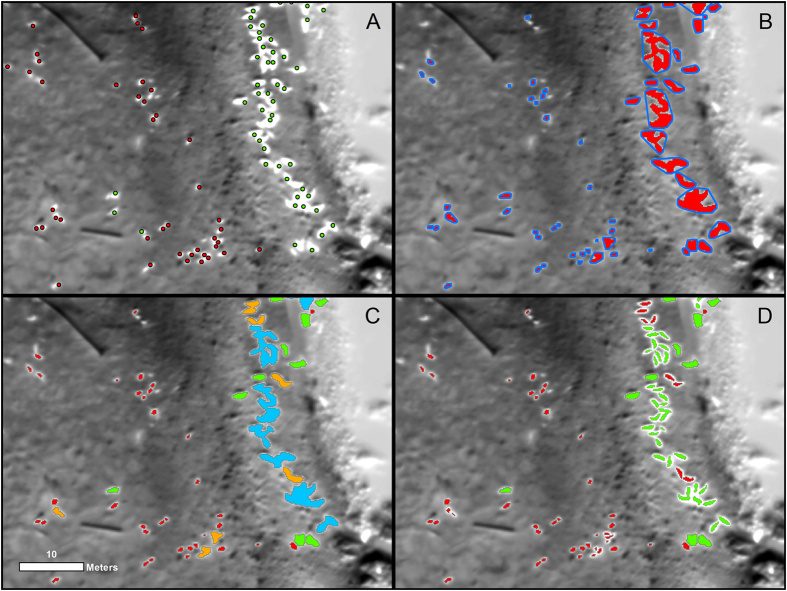
(**A**) Thermal imagery with overlaid human-identified seal points (red = YOY, green = adult). (**B**) Red seal polygons outlined by blue convex hulls. (**C**) Tier 1 model classification of seals. Blue polygons are adult aggregations, orange polygons are YOY aggregations, green polygons are individual adults and red polygons are individual YOYs. (**D**) Aggregation polygons after high pass filtering, broken up into individual adults and YOYs. This map was created with ArcMap GIS software (version 10.4.1, Esri Inc.).

All pixels in the Hay Island thermal imagery greater than or equal to 9 °C were selected. We chose this value after visually estimating a lower thermal boundary for seal identification that excluded warmer landscape features. Selected pixels were then converted into polygons (Panel B, Fig. 3) and the area and average temperature of each polygon was recorded.

Because seals touching one another in aggregations resulted in single polygons with irregular shapes, we built convex hulls around all polygons (Panel B, Fig. 3). A ratio between the area of the original polygon and the area of its convex hull was appended to each original polygon, serving as a basis to discriminate individual seals from seal aggregations.

Resulting polygons were then classified as individual YOYs, individual adults, YOY aggregations or adult aggregations (Panel C, Fig. 3). Classification parameters (Table 1, “complex model”) were developed from a spatially-referenced dataset of YOYs and adults that were identified manually from UAS thermal and RGB imagery using the program ITag. YOY classification was based on polygon size as well average temperature, as YOYs had smaller planar areas and tended to be cooler than adults. Adult classification was based upon polygon size, temperature and polygon shape; adults tended to be warmer and larger than YOYs and had smoother shapes than aggregations. Classification of YOY aggregations were based on polygon size and shape. The YOY aggregations tended to be irregularly shaped, smaller than adult aggregations and not thermally distinct. Adult aggregations were classified based on polygon size and shape; adult aggregations were larger than YOY aggregations and irregularly shaped compared to large individuals.

Aggregation polygons were additionally processed with a high pass filter. High pass filters are neighborhood functions that accentuate the differences between pixels and are effective at detecting edges (a mathematical description of the filter can be found in appendix A of the Supplementary Materials). The filter separated aggregation polygons into clusters of pixels representing individual seals, which were then converted to polygons representing individual seals (Panel D, Fig. 3). All polygons derived from adult aggregations were assumed to be adult seals, and all polygons derived from YOY aggregations were assumed to be YOY. Total seal counts were generated by summing the individual YOY and individual adult polygons.

We also tested the above methods in a separate estimate of the grey seal populations at Saddle Island. Size and shape-based classification parameters trained at Hay Island were based on spatially-explicit 2-dimensional profiles of seals, and we re-applied those parameters at Saddle Island, only modifying the lower thermal detection threshold to 5.5 °C to avoid overlap with ambient landscape temperature. We tested two different variants (simplified and complex) of the model on Saddle Island. The simplified version used only planer area and polygon shape to discriminate individual YOY and adult polygons (Table 1 “simplified model”), dropping the temperature parameter. The complex variant retained temperature as a classification parameter for individual YOY and adult polygons, with temperature parameters from Table 1 offset by the difference between Hay Island and Saddle Island lower thermal thresholds (−3.5 °C). To minimize bias, the analyst developing and testing the model did not view the Saddle Island thermal dataset until modeling on Hay Island was completed.

For accuracy assessments at Hay and Saddle Island, model-predictions were compared to the human-identified ITag points. To correct for minor GPS error, ITag points within 0.5 m of prediction polygons were moved within the extent of prediction polygons. We then spatially joined ITag points with the model-generated prediction polygons, recording any points that overlapped with polygons.

### Data Availability

All data used in analysis including human point counts of seals, thermal indices and orthomosaics are available for viewing and download at http://seamap.env.duke.edu/dataset/1462.

## Results

### UAS Imagery

**Figure 4:**
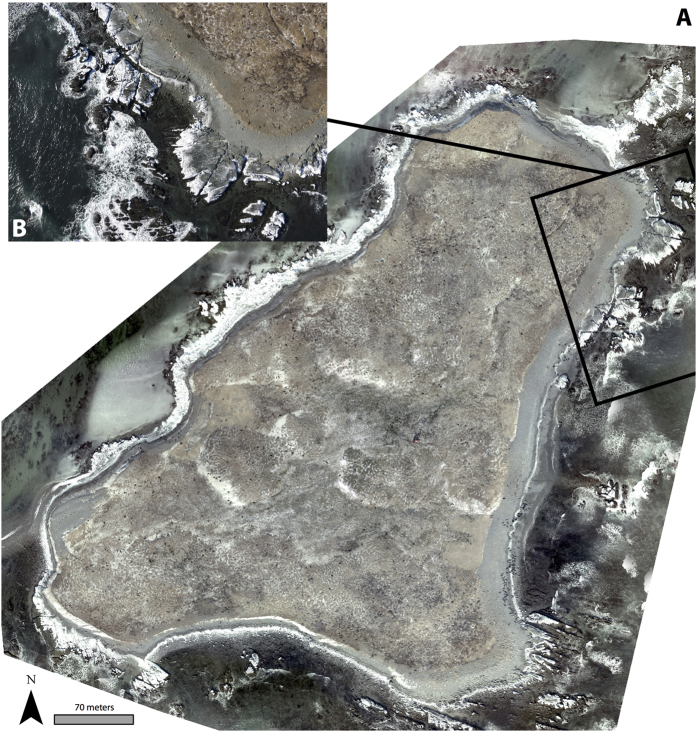
An RGB orthomosaic (**A**) and a representative individual RGB image (**B**) of the grey seal colony at Hay Island, NS Canada. This footrprint of the individual image is projected onto the orthomosaic, providing a detailed view of adult and YOY grey seals and the habitats surveyed (rock, beach and frozen ground). This map was created with ArcMap GIS software (version 10.4.1, Esri Inc.).

**Figure 5:**
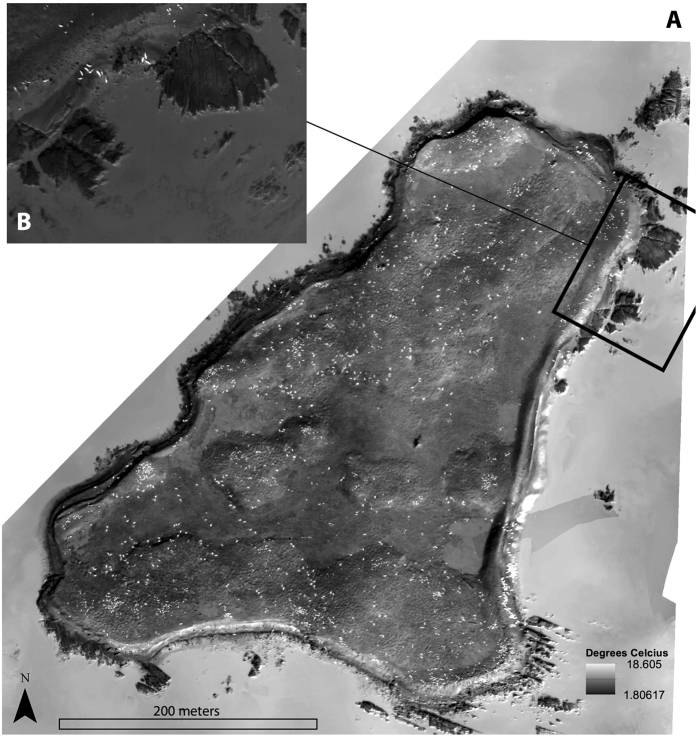
A thermal infrared spatial index map (**A**) and a representative individual thermal infrared image (**B**) of the grey seal colony at Hay Island, NS Canada. This footprint of the individual image is projected onto the spatial index, providing a detailed view of adult and YOY grey seals and the habitats surveyed (rock, beach and frozen ground). This map was created with ArcMap GIS software (version 10.4.1, Esri Inc.).

The RGB orthomosaic for Hay Island and a sample single image from the UAS are presented in Fig. 4a and b respectively. Similarly, the temperature index map for Hay Island, and a sample thermal image is presented in Fig. 5a and b respectively. The orthomosaics demonstrate that both RGB and thermal surveys covered the entire island colony, and each sample image reveals individual adult and YOY grey seals.

### Seal Counts

Human-conducted counts using ITag were very similar to seal detection model estimates (Table 2). Total seal counts between the detection model and human guided methods were within ~95% of one another at Hay Island and ~98% of one another at Saddle Island and did not differ between simple and complex classification. The model detected ~91% of the seals counted by humans at Hay Island and ~96% at Saddle Island, performing better at the prediction site than the training site. The two methods were also very similar in their sub counts of YOYs and adults, with greatest conformance at Saddle Island using simplified classification. Automated detection rarely mis-identified landscape features as seals, and most of the seals the model detected that were not corroborated in ITag appeared to be seals that human analysts overlooked. The model slightly undercounted seals when compared to traditional methods, missing a small number of animals that were below the chosen lower temperature detection threshold.

The seal detection model predicted individual YOYs with a high level of accuracy at both locations, but was closer to human-generated counts at Saddle Island (Table 3) regardless of whether simple or complex classification was used. The model classified some thermal signatures as YOYs that humans did not. Upon investigation, most these additional detections appeared to be seals and not misclassified landscape features. The rate of true misclassification appeared higher at Hay Island where ambient landscape temperatures were warmer. Likewise, the model failed to detect some human-identified YOYs that were below the model’s temperature-detection threshold, which was more prevalent at Hay Island. At both study sites, the number of additional seals detected by the model and the seals the model missed nearly negated one another.

The model predicted individual adults at a true positive rate of ~81% at both sites when simplified classification was applied on Saddle Island (Table 3). When misclassification occurred, the model most commonly interpreted larger human-identified YOYs as adults. Using complex classification, the true positive rate fell to ~67%, accentuating the tendency for larger, warmer human-identified YOYs to be misclassified as adults. There were very few human-identified adults that were entirely missed by the model; At Hay Island there were 11 adults that were not classified by any seal prediction polygons and at Saddle Island there were 2.

## Discussion

This paper provides a compelling example of how small UAS and easy-to-use, automated GIS workflows can be used to enhance wildlife surveys. The use of thermal imagery for counting pinnipeds has been used previously^25^, although early instruments did not have the sensitivity required to discriminate many seals from the background^31^. Our approach may be especially useful to assess new and growing colonies of grey seals as they reoccupy portions of the traditional range in the Western North Atlantic^26^,^29^. As these animals recover and interact with human activities (e.g. ref. 31), it is increasingly important to understand trends in their abundance. Small UAS also provide detailed information on the habitats found at gray seal colonies and these data are crucial for managing colony interactions with human use patterns at those locations^26^.

The seal detection model recorded a similar number of grey seals in UAS-collected thermal imagery as human generated counts in ITag, and effectively applied parameters from a training site to a spatially and temporally separate prediction site. The model compared better to human counts at the prediction site (Saddle Island) where ambient landscape temperatures were lower, allowing for better contrast between seals and the environment. Discrepancies between human and model counts were due to humans overlooking YOYs, the lower thermal detection boundary of the model, size/temperature similarities between large YOYs and small adults, and an inability to distinguish between YOY aggregations and pup/mother pairs.

Much of the difference in counts between the two methods was due to the detection of individual YOYs. Some of the apparent false positives (Table 3, Not Detected by Humans) were pups that human analysts failed to detect. Inappropriate image value scaling, line symbology and landscapes with different aspects and substrate types can cause smaller, colder seal pups appear to a human analyst like landscape features. Conversely, the model failed to detect YOYs below the specified lower thermal boundaries while human analysts could detect colder YOYs if they were laying out on contrasting landscape (Table 3, YOYs not detected by model). These false positives may also be the result of humans detecting dead YOYs that deviate from background temperatures. While uncommon, in some instances the model classified small patches of warmer landscape as YOYs, and in many cases these small patches were individual pixels. This phenomenon was more prevalent at Hay Island, where the ambient landscape temperature overlapped with the thermal signature of seals in some areas. Selecting a higher temperature detection threshold could help avoid these false positives, but threshold selection is a trade-off between detecting seals and making commission errors. A winnowing filter was also not an appropriate solution to this problem because, in many instances at Hay Island, polygons consisting of a single pixel correctly represented YOYs.

The above false positives can be reduced or eliminated by collecting UAS thermal images during early dawn, reducing ambient landscape temperature and increasing the contrast between seals and landscape. This allows for selection of a colder lower thermal boundary in the model, which detects more YOYs without miss-identifying warmer landscape features as seals. Improving thermal contrast can also eliminate the need to mask out warm ambient landscape that overlaps with seals during analysis, which can make it impossible to detect some seals. However, it is important to consider seal haul-out behavior when planning dawn flights to avoid grossly underestimating populations, and also to allow enough light for humans to spot-check model results using RGB imagery^32^.

Most remaining discrepancies between model and human methods were related to the binning of seals into adult or YOY classes. For instance, individuals derived from adult or YOY aggregation polygons were automatically binned into the demographic class indicated by their aggregation polygon. While adult aggregations nearly only consisted of adult seals, YOY aggregation polygons sometimes included adult females adjacent to their newborn pups. In these cases, the model incorrectly identified adults as YOYs. These false positives could be eliminated with a second round of object size analysis.

While the model’s simple and complex classification approaches recorded identical total seal counts, they binned adults and YOY differently. Complex classification took temperature into account to correctly parse larger YOY and smaller adults, but detected adults worse than simplified classification when human methods were used as a benchmark. The 3.5 °C offset applied to the complex classification parameters at Saddle Island was adopted from the difference in user-selected lower temperature thresholds between the two study datasets. It is possible this coarse offset did not account for seals’ responses to the lower ambient temperature at Saddle, including the potential for YOYs and adults to react to colder ambient temperature differently. Additionally, the temperature difference between YOYs and adults may be sensitive to immediate thermal landscape that the model was not designed to detect. Considering these uncertainties and the value of automated detection with minimal setup time, the parsimonious classification scheme is preferable and allows for a more universal application of the model.

The detection model in this study has a logical flow applicable to other homeotherms that gather in large numbers on thermally-contrasting landscape, including colonial seabirds such as penguins, social ungulates like horses or deer, some primate species and many other pinnipeds. Our polygon/convex hull ratio and high-pass filter combination is effective at counting animals within dense aggregations where thermal signatures overlap, which has been a challenge in past computer vision studies. While beyond the scope of the present study, longer-term projects that adopt our approach may develop methods for predicting the accuracy of other detection methods (i.e. human-guided counting), or to correct for misclassification error within a larger statistical model. However, the model is not suitable for analysis of imagery containing multiple species and, in this study, would likely misclassify any homeotherm with a body size similar to a seal. As such, this approach is suitable for enumeration of single species colonies where the target species represents the vast majority of animals present. The model is also sensitive to large shifts in ground sampling resolution. Our analysis is performed on corrected, spatially rectified imagery and individual seal classification relies largely on the planar area of objects, which should remain relatively constant with resolution shift. However, aggregation detection relies on convex hull ratios, which may be more sensitive to large resolution changes.

Automated image classification is often considered a means to save time and costs associated with human-driven analysis^21^. However, these approaches are of limited utility if models must be re-trained for each new dataset, or require extensive set up time. Our model applies parameters from a single training site to a spatially and temporally separate prediction site, estimating populations with accuracy meeting or exceeding traditional human-guided methods. Specifically, machine-based counts of animals can help eliminate individual variability in counts conducted by different people. Our “plug and play” approach is parsimonious, requiring only a site-specific temperature detection threshold as an input. This value can be chosen after 10–15 minutes of visual investigation of a thermal orthomosaic. Our workflow could be easily applied to a range of sites, especially if the sampling method is conserved.

Automated image classification models like the one in this study synergize well with UAS. Appropriate use of UAS can output more precise counts than traditional methods^18^ and can yield cost savings. Additionally, UAS assessment of marine vertebrate populations can reduce human risk, and a recent review of job-related mortalities of wildlife biologists revealed that a significant proportion arose from aviation accidents^6^. This type of risk is amplified when working in coastal regions or over the water. The combination of automated image classification and UAS in this study presents a compelling argument for a decrease in the cost of wildlife assessments, a reduction in analyst time and minimization of risk to human surveyors.

## Additional Information

**How to cite this article**: Seymour, A. C. *et al*. Automated detection and enumeration of marine wildlife using unmanned aircraft systems (UAS) and thermal imagery. *Sci. Rep.* **7**, 45127; doi: 10.1038/srep45127 (2017).

**Publisher's note:** Springer Nature remains neutral with regard to jurisdictional claims in published maps and institutional affiliations.

## Supplementary Material

Supplementary Appendix A

## Figures and Tables

**Table 1:** Classification parameters for the seal detection model.

Polygon class	Classification parameters
Individual Juvenile	**Complex Model:** Polygon Area ≤ 0.85 m^2^ and Mean Temperature <10 °C OR Polygon Area <0.65 m^2^**Simplified Model:** Polygon Area ≤ 0.85 m^2^
Individual Adult	**Complex Model:** Polygon Area >0.65 m^2^ and ≤3.5 m^2^ and Polygon/Convex Hull Ratio >0.8 and Mean Temperature >10 °C OR Polygon Area >0.85 m^2^ and ≤3.5 m^2^ and Polygon/Convex Hull Ratio >0.8**Simplified Model:** Polygon Area >0.85 m^2^ and Polygon Area ≤ 3.5 m^2^ and Polygon/Convex Hull Ratio >0.8
Juvenile Aggregation	Polygon Area >0.65 m^2^ and <0.85 m^2^ and Polygon/Convex Hull Ratio <0.75 OR Polygon Area >0.85 m^2^ and <3.5 m^2^ and Polygon/Convex Hull Ratio <0.8
Adult Aggregation	Polygon Area >3.5 m^2^ and Polygon/Convex Hull Ratio <0.8

**Table 2:** Comparison of computer vision and human-generated seal count estimates.

Seal Count Estimates					
Detection Model				ITag (human-generated)	
Category	Hay Island	Saddle Island (Simple)	Saddle Island (Complex)	Hay Island	Saddle Island
Total Juveniles	1652	648	592	1743	660
Total Adults	536	246	302	568	253
Total Seals:	**2188**	**894**		**2311**	**913**

**Table 3:** Accuracy assessment for model-generated individual YOY and adult prediction polygons.

Result	Description	Hay Island	Proportion	Saddle (Simple)	Proportion	Saddle (Complex)	Proportion
YOY Prediction Accuracy							
True Positive	NA	1329	0.804	566	0.871	515	0.870
False Positive	Confused with Adult	105	0.064	33	0.051	30	0.051
False Positive	Confused with Juvenile Aggregation	48	0.029	3	0.005	3	0.005
False Positive	Confused with Adult and Juvenile Pair	0	0.000	3	0.005	3	0.005
False Positive	Not Detected by Humans	170	0.103	43	0.066	41	0.069
Human-Identified Juveniles Not Detected By Model		215		39			
Adult Prediction Accuracy							
True Positive	NA	436	0.813	199	0.809	202	0.669
False Positive	Confused with Juvenile	68	0.127	23	0.093	75	0.248
False Positive	Confused with Juvenile Aggregation	26	0.049	9	0.037	9	0.030
False Positive	Confused with Adult and Juvenile Pair	6	0.011	13	0.053	13	0.043
False Positive	Not Detected by Humans	0	0.000	2	0.008	3	0.010
Human-Identified Adults Not Detected By Model		11		2			

Model estimates were assessed against human-identified ITag counts, so that “true positives” indicate prediction polygons that overlap with matching ITag points.
