# Supplementary material for: Automated detection and enumeration of marine wildlife using unmanned aircraft systems (UAS) and thermal imagery
Source: Sci Rep. 2017 Mar 24;7:45127. doi: 10.1038/srep45127 (PMC5364474; doi:10.1038/srep45127)
Supplement: Supplementary Appendix A [file srep45127-s1.pdf]

Automated detection and enumeration of marine wildlife using unmanned aircraft systems (UAS) and thermal imagery

A. C. Seymour[1]*, J. Dale[1], M. Hammill[2], P. N. Halpin[1] and D. W. Johnston[1]

[1] Division of Marine Science and Conservation, Nicholas School of the Environment, Duke University Marine Laboratory. 135 Duke Marine Lab Rd. Beaufort, NC 28516

[2] Peches et Oceans Canada/Fisheries and Oceans Canada. Institut Maurice-Lamontagne/Maurice Lamontagne Institute. C.P. 1000/P.O. Box 1000 850 Route de la Mer Mont-Joli, QC G5H 3Z4

Correspondance to alexander.c.seymour@duke.edu

Appendix A: Automated Detection Model Script

```
# Grey Seal Detection and Enumeration Model
# Designed for use in ArcMap and arcpy-enabled applications like Pythonwin
# ------------------------------------------------------------------------------------------------
# Devised, Coded and Tested by Alexander Cy Seymour
# Created: September, 2016
# Have Questions? Email acs72@duke.edu
# ------------------------------------------------------------------------------------------------

# Import arcpy module and load licenses
import arcpy, sys
from arcpy import env
from arcpy.sa import *
arcpy.CheckOutExtension("Spatial")
arcpy.env.overwriteOutput = True

#Set Workspaces ***IMPORTANT, READ COMMENT BELOW***
Scratch = sys.argv[1]
arcpy.env.scratchWorkspace = Scratch
Data = sys.argv[2]
arcpy.env.workspace = Data
#The user must select two directories to use a scratch and primary workspace
#for intermediate and final outputs. The scratch workspace must be a folder named
#"Scratch" and the primary workspace must be a folder named "Data". Both of these
#folders must be in the same directory as this script.

#Set Variables and Inputs
ThermalIndex = sys.argv[3]
#User selects a thermal index (raster filetype). This raster must be placed in the
#primary workspace (the "Data" folder).

TempSelect = sys.argv[4]
#user inputs a lower thermal threshold representing the
#temperature that YOY seals can be found on the thermal index.

#Set Geoprocessing Environments
```

```
arcpy.env.outputCoordinateSystem = ThermalIndex
arcpy.env.snapRaster = ThermalIndex

#Processesing Workflow Begins

# Process: Con (6)
arcpy.gp.Con_sa(ThermalIndex, "1", "Scratch\\ConOut.tif", "", "value > " + TempSelect)
#Selects all the cells with a temperature index greater than or equal to the
#user's selection.

# Process: Region Group
arcpy.gp.RegionGroup_sa("Scratch\\ConOut.tif", "Scratch\\RegionGroup.tif", "EIGHT", "WITHIN",
"NO_LINK", "")
#This step prevents isolated pixels directly adjacent and diagonal to other pixel clusters
#from being broken into separate polygons in the following raster to polygon step.

# Process: Raster to Polygon (5)
arcpy.RasterToPolygon_conversion("Scratch\\RegionGroup.tif", "Scratch\\RtoP.shp", "NO_SIMPLIFY",
"Value")
#Vectorizes all pixels slected by the previous step and converts them to polygons.

# Process: Dissolve
arcpy.Dissolve_management("Scratch\\RtoP.shp", "Scratch\\MainPoly.shp", "GRIDCODE", "",
"MULTI_PART", "DISSOLVE_LINES")
#Reorganizes the large single polygon created in the previous step into a multipart
#polygon, so that each polygon part can have its own attributes. This step is made
#necessary due to the earlier use of the Region Group tool.

# Process: Add Geometry Attributes
arcpy.AddGeometryAttributes_management("Scratch\\MainPoly.shp", "AREA", "",
"SQUARE_METERS", "")
#Adds area atributes to all polygon pieces.

# Process: Minimum Bounding Geometry
arcpy.MinimumBoundingGeometry_management("Scratch\\MainPoly.shp", "Scratch\\ConvexHulls.shp",
"CONVEX_HULL", "NONE", "", "MBG_FIELDS")
#Builds new convex hull polygons around all of the original polygon pieces.

# Process: Add Geometry Attributes (6)
arcpy.AddGeometryAttributes_management("Scratch\\ConvexHulls.shp", "AREA", "",
"SQUARE_METERS", "")
#Attaches an area attribute to each of convex hull polygons.

# Process: Join Field
arcpy.JoinField_management("Scratch\\MainPoly.shp", "GRIDCODE", "Scratch\\ConvexHulls.shp",
"GRIDCODE", "POLY_AREA")
#Joins the convex hull polygon areas to the attribute tables of the original polygons
#they were built around.

# Process: Zonal Statistics as Table
```

```
arcpy.gp.ZonalStatisticsAsTable_sa("Scratch\\MainPoly.shp", "GRIDCODE", ThermalIndex,
"Scratch\\ZonalStats", "DATA", "MEAN")
#Averages the values of the thermal index under each polygon and puts the results in
#a table.

# Process: Join Field (3)
arcpy.JoinField_management("Scratch\\MainPoly.shp", "GRIDCODE", "Scratch\\ZonalStats",
"GRIDCODE", "Mean")
#Appends the mean thermal index results to the attribute table of the appropriate
#polygons.

# Process: Add Field (2)
arcpy.AddField_management("Scratch\\MainPoly.shp", "P_C_Ratio", "DOUBLE", "7", "4", "", "",
"NULLABLE", "NON_REQUIRED", "")
#Adds a field for the polygon/convex hull ratios to be calculated in.

# Process: Calculate Field (2)
arcpy.CalculateField_management("Scratch\\MainPoly.shp", "P_C_Ratio", "[POLY_AREA]/
[POLY_ARE_1]", "VB", "")
#Calculates the polygon/convex hull ratio and puts the results in the "P_C_Ratio" column
#of the attribute table. Note that "POLY_ARE_1" refers to the column "POLY_AREA" added in
#the previous Join Field process. Its name was automatically changed when it was joined to
#"MainPoly.shp" because "MainPoly.shp" already had a field called "POLY_AREA".

# Process: Select
arcpy.Select_analysis("Scratch\\MainPoly.shp", "Data\\IndividualYOY.shp", "\"POLY_AREA\" <=
0.85")
#Runs Selection critera on the polygons and classifies those that meet the critera as
#individual YOY. Critera Shown are for the simplified classification scheme. Critera for
#the complex scheme would be:
#"\"POLY_AREA\" <= 0.85 AND \"MEAN\" < 6.5 OR \"POLY_AREA\" < 0.65"
#Note that the MEAN < 6.5 degrees value is set for the Saddle Island dataset and was offset
#from parameters trained at Hay Island. If you choose to use the complex classification
#scheme, first review the methods section of the manuscript to properly offset this value
#for your new dataset.

# Process: Select (2)
arcpy.Select_analysis("Scratch\\MainPoly.shp", "Data\\IndividualAdult.shp", "\"POLY_AREA\" >0.85
AND \"POLY_AREA\" <=3.5 AND \"P_C_Ratio\" >0.8")
#Runs Selection critera on the polygons and classifies those that meet the critera as
#individual adults. Critera Shown are for the simplified classification scheme. Critera for
#the complex scheme would be:
#"\"POLY_AREA\" > 0.65 AND \"POLY_AREA\" <= 3.5 AND \"P_C_Ratio\" > 0.8 AND \"MEAN\" >
6.5
#OR \"POLY_AREA\" > 0.85 AND \"POLY_AREA\" <= 3.5 AND \"P_C_Ratio\" > 0.8"
#Note that the MEAN > 6.5 degrees value is set for the Saddle Island dataset and was offset
#from parameters trained at Hay Island. If you choose to use the complex classification
#scheme, first review the methods section of the manuscript to properly offset this value
#for your new dataset.

# Process: Select (3)
```

```
arcpy.Select_analysis("Scratch\\MainPoly.shp", "Scratch\\AdultPiles.shp", "\"POLY_AREA\" > 3.5 AND
\"P_C_Ratio\" < 0.8")
#Runs Selection critera on the polygons and classifies those that meet the critera as adult
#aggregation polygons.

AdultPileDesc = arcpy.Describe("Scratch\\AdultPiles.shp")
AdultPileExtent = ("{0} {1} {2} {3}".format(AdultPileDesc.extent.XMin, AdultPileDesc.extent.YMin,
AdultPileDesc.extent.XMax, AdultPileDesc.extent.YMax))
#Records the extent of the AdultPiles shapefile so that it can be input into the clip
#process below.

# Process: Clip (2)
arcpy.Clip_management(ThermalIndex, AdultPileExtent, "Scratch\\ThermAClip.tif",
"Scratch\\AdultPiles.shp", "-3.402823e+038", "ClippingGeometry", "NO_MAINTAIN_EXTENT")
#Isolates the thermal index under the adult aggregation polygons in preparation for a
#high pass filter.

# Process: Filter (2)
arcpy.gp.Filter_sa("Scratch\\ThermAClip.tif", "Scratch\\AdultHigh.tif", "HIGH", "NODATA")
#Runs a high pass filter on the isolated areas of the thermal index.
#The filter runs a neighborhood function on each pixel of the input raster.
#A new, normalized value is calculated for the center pixel of each neighborhood.
#This is done by multiplying the neighborhood by the following values:
# -0.7 -1.0 -0.7
# -1.0  6.8 -1.0
# -0.7 -1.0 -0.7
#Then, the results are summed and the value given to the center cell. This process
#is carried out for each cell in the input raster.
#This is a standard tool in the spatial analyst toolbox in ESRI's ArcMap software.
#More information can be found at:
#http://resources.arcgis.com/en/help/main/10.1/index.html#//009z000000r5000000
#or by searching the web for "arcmap high pass filter".
#The results of the filter create high value pixels on the edges of each seal
#in a given aggregation polygon. These pixels are much higher in value than their
#neighbors and can easily be thresholded and vectorized. The results of this filter
#are normalized so that edges across different datasets will have the same values.

# Process: Con
arcpy.gp.Con_sa("Scratch\\AdultHigh.tif", "1", "Scratch\\AggCon.tif", "", "\"value\" > 1")
#Selects the output pixels from the high pass filter that represent the edges of seals.

# Process: Raster to Polygon
arcpy.RasterToPolygon_conversion("Scratch\\AggCon.tif", "Scratch\\RtoPFilteredAdults.shp",
"NO_SIMPLIFY", "VALUE")
#Vectorizes the selected pixels from the previous step, creating a new polygon feature
#of individual adults isolated from their aggregations.

# Process: Add Geometry Attributes (2)
arcpy.AddGeometryAttributes_management("Scratch\\RtoPFilteredAdults.shp", "AREA", "",
"SQUARE_METERS", "")
#Appends each of the polygons created in the previous step with an area attribute.
```

```
# Process: Select (4)
arcpy.Select_analysis("Scratch\\RtoPFilteredAdults.shp", "Data\\AggregationAdults.shp",
"\"POLY_AREA\">0.15")
#Removes very small polygons from the aggregation adults, as these are usually the result
#of ambient landscape pixels within the aggregation polygon extents that were accentuated
#by the high pass filter.

# Process: Select (5)
arcpy.Select_analysis("Scratch\\MainPoly.shp", "Scratch\\YOYPiles.shp", "\"POLY_AREA\" > 0.65
AND \"POLY_AREA\" < 3.5 AND \"P_C_Ratio\" < 0.75 OR \"POLY_AREA\" > 0.85 AND
\"POLY_AREA\" < 3.5 AND "\"P_C_Ratio\" < 0.8")
#Runs Selection critera on the polygons and classifies those that meet the critera as
#YOY aggregation polygons.

YOYPileDesc = arcpy.Describe("Scratch\\YOYPiles.shp")
YOYPileExtent = ("{0} {1} {2} {3}".format(YOYPileDesc.extent.XMin, YOYPileDesc.extent.YMin,
YOYPileDesc.extent.XMax, YOYPileDesc.extent.YMax))
#Records the extent of the YOYPiles shapefile so it can be input into the clip
#process below.

# Process: Clip (3)
arcpy.Clip_management(ThermalIndex, YOYPileExtent, "Scratch\\ThermYClip.tif",
"Scratch\\YOYPiles.shp", "-3.402823e+038", "ClippingGeometry", "NO_MAINTAIN_EXTENT")
#Isolates the thermal index under the YOY aggregation polygons in preparation for a
#high pass filter.

# Process: Filter (3)
arcpy.gp.Filter_sa("Scratch\\ThermYClip.tif", "Scratch\\YOYHigh.tif", "HIGH", "NODATA")
#Runs a high pass filter on the isolated areas of the thermal index.
#The filter runs a neighborhood function on each pixel of the input raster.
#A new, normalized value is calculated for the center pixel of each neighborhood.
#This is done by multiplying the neighborhood by the following values:
# -0.7 -1.0 -0.7
# -1.0  6.8 -1.0
# -0.7 -1.0 -0.7
#Then, the results are summed and the value given to the center cell. This process
#is carried out for each cell in the input raster.
#This is a standard tool in the spatial analyst toolbox in ESRI's ArcMap software.
#More information can be found at:
#http://resources.arcgis.com/en/help/main/10.1/index.html#//009z000000r5000000
#or by searching the web for "arcmap high pass filter".
#The results of the filter create high value pixels on the edges of each seal
#in a given aggregation polygon. These pixels are much higher in value than their
#neighbors and can easily be thresholded and vectorized. The results of this filter
#are normalized so that edges across different datasets will have the same values.

# Process: Con (3)
arcpy.gp.Con_sa("Scratch\\YOYHigh.tif", "1", "Scratch\\YOYAggCon.tif", "", "\"value\" > 1")
#Selects the output pixels from the high pass filter that represent the edges of seals.
```

# Process: Region Group (2)
arcpy.gp.RegionGroup_sa("Scratch\\YOYAggCon.tif", "Scratch\\YOYRegGroup.tif", "EIGHT", "WITHIN", "ADD_LINK", "")
#This step prevents isolated pixels directly adjacent and diagonal to other pixel
#clusters from being broken into separate polygons in the following raster to polygon step.

# Process: Raster to Polygon (2)
arcpy.RasterToPolygon_conversion("Scratch\\YOYRegGroup.tif", "Scratch\\YOYAggRtoP.shp", "NO_SIMPLIFY", "VALUE")
#Vectorizes the selected pixels from the previous step, creating a new polygon feature
#of individual YOY isolated from their aggregations.

# Process: Dissolve (2)
arcpy.Dissolve_management("Scratch\\YOYAggRtoP.shp", "Data\\AggregationYOY.shp", "GRIDCODE", "","MULTI_PART", "DISSOLVE_LINES")
#Reorganizes the large single polygon created in the previous step into a multipart polygon,
#so that each polygon part can have its own attributes. This step is made necessary due to the
#earlier use of the Region Group tool.

#To get total number of seals, add the polygons from "IndividualAdults.shp", "IndividualYOY.shp",
#"AggregationAdults.shp" and "AggregationYOY.shp". This can be done simply looking at the attribute
#table of each shapefile and looking at the feature count at the botton of the table UI.
